# Highly Efficient and Scalable Separation of Semiconducting Carbon Nanotubes via Weak Field Centrifugation

**DOI:** 10.1038/srep26259

**Published:** 2016-05-18

**Authors:** Wieland G. Reis, R. Thomas Weitz, Michel Kettner, Alexander Kraus, Matthias Georg Schwab, Željko Tomović, Ralph Krupke, Jules Mikhael

**Affiliations:** 1 Carbon Materials Innovation Center (CMIC), BASF SE, 67056 Ludwigshafen, Germany; 2 FET Systems, BASF SE, 67056 Ludwigshafen, Germany; 3 InnovationLab GmbH, Heidelberg, Germany; 4 Advanced Materials & Systems Research, BASF Construction Solutions GmbH, 83308 Trostberg, Germany; 5 Department of Materials and Earth Sciences, Technische Universität Darmstadt, Darmstadt, Germany; 6 Material Physics Research, BASF SE, 67056 Ludwigshafen, Germany

## Abstract

The identification of scalable processes that transfer random mixtures of single-walled carbon nanotubes (SWCNTs) into fractions featuring a high content of semiconducting species is crucial for future application of SWCNTs in high-performance electronics. Herein we demonstrate a highly efficient and simple separation method that relies on selective interactions between tailor-made amphiphilic polymers and semiconducting SWCNTs in the presence of low viscosity separation media. High purity individualized semiconducting SWCNTs or even self-organized semiconducting sheets are separated from an as-produced SWCNT dispersion via a single weak field centrifugation run. Absorption and Raman spectroscopy are applied to verify the high purity of the obtained SWCNTs. Furthermore SWCNT - network field-effect transistors were fabricated, which exhibit high ON/OFF ratios (10^5^) and field-effect mobilities (17 cm^2^/Vs). In addition to demonstrating the feasibility of high purity separation by a novel low complexity process, our method can be readily transferred to large scale production.

Owing to their exceptional electronic properties single-walled carbon nanotubes (SWCNTs) have gained significant attention for use in the microelectronics industry^1^. One of the largest challenges while downscaling the lateral dimensions of transistors is to minimize their power consumption per logic operation. Here, SWCNTs offer a distinct advantage over inorganic materials^2^. This is why they have become a promising candidate to potentially replace silicon. However, the limiting factor for SWCNTs is that all large-scale production processes yield a statistical mix of semiconducting and unwanted metallic SWCNTs as well as amorphous carbon. The drawback of recently developed production routes^3^,^4^,^5^,^6^ yielding narrow chirality distributions is not only the limited scalability but also the still insufficient purity for microelectronic applications. The high integration density of logic circuits requires nanotube inks to have a very high semiconducting purity^7^, since metallic nanotubes would lead to device failure. Over the past decade, considerable effort has therefore been dedicated to developing post-synthesis methods for the isolation of high-purity semiconducting nanotubes from the as-synthesized mix. The approaches reported in literature include density gradient ultracentrifugation (DGU)^8^,^9^,^10^, use of electric fields^11^,^12^, selective polymer wrapping^13^,^14^,^15^,^16^, spontaneous phase separation^17^,^18^,^19^, and gel-chromatography^20^,^21^. Although these known separation processes have partially proven to be capable of providing high purity or nearly single-chirality semiconducting SWCNTs, most processes suffer from low yields, elaborate multi-step procedures or rely on expensive equipment (e.g. ultracentrifuges or sophisticated chromatographic columns), which today greatly hampers the commercial competitiveness of SWCNTs.

In this study, we demonstrate for the first time that highly efficient purification of semiconducting SWCNTs is possible in one separation step and using only conventional centrifugation, i.e. weak field centrifugation (WFC). With our method, not only individualized semiconducting tubes can be isolated but also self-organized freestanding sheets with very high contents of semiconducting SWCNT species are obtained. We have proven the excellent quality of the separated semiconducting SWCNTs via spectroscopic tools as well as by field-effect transistor measurements. Our method offers the potential to be transferred to larger process dimensions without the use of expensive and hardly scalable laboratory equipment.

In contrast to the separation approach based on selective polymer wrapping in organic solvents^14^,^22^, the WFC approach in aqueous media presented in this work is enabled by the combination of two key elements: comb polymers with tailored amphiphilic properties and low viscosity aqueous solutions of heavy liquids. The comb polyarylether (PAE) polymers have a strong amphiphilic character and thus act as highly potent dispersants for SWCNTs. Low viscosity aqueous heavy liquid solutions, such as the non-toxic sodium or lithium polytungstate (SPT, LST), are applied for the first time as separation media to SWCNT purification.

The general structure of the PAE polymer used in this work can be found in Fig. 1a. For the detailed synthesis see Supplementary Section 1. It has a hydrophobic backbone based on benzene rings which are substituted with hydrophobic dodecyl side chains as well as hydrophilic polyethylenglycol (PEG) side chains and phenoxyethanolphosphate groups. Our experiments show that this polymer has a strong affinity to carbon nanotubes (CNTs) believed to be promoted by π–π interactions with its aromatic backbone^23^ and the hydrophobic interaction originating from the dodecyl side chains. The hydrophilic PEG side chains and the anionic phenoxyethanolphosphate moieties ensure an electrosteric stabilization of the individualized tubes and strongly prevent their re-agglomeration^24^. In fact, due to strong van-der-Waals forces, aqueous CNT dispersions always contain bundles of metallic and semiconducting tubes. Since the majority of separation techniques require individualized SWCNTs, ultracentrifugation is widely used prior to the actual separation stage to remove the bundled tubes and catalytic impurities^17^,^21^. This additional step, not required in our case, is associated with the loss of semiconducting tubes and thus reduces the yield to a large extent.

The experimental study of the separation method is schematically depicted in Fig. 1. SWCNTs were dispersed by horn sonication in an aqueous solution of the polymeric dispersant (Fig. 1a). Without any pre-separation, the dispersion, set to pH 4, was then loaded on top of an SPT column within a centrifugation vessel (Fig. 1b). This is followed by a single centrifugation step employing a weak centrifugal field of about 10,000 × *g*. Photographs of different final separation states are depicted in Fig. 1c. They represent the separation results from the procedure described above when performed under three different conditions. When the initial SPT solution is neutralized, i.e. set to pH 7, we observed after 25 h of WFC that the SWCNTs were only separated from the carbonaceous impurities which have accumulated at the bottom of the vessel (Fig. 1c-1). Surprisingly, WFC under acidic conditions (~pH 1.8) leads to a different separation result. A single (green-blue) band was isolated in the upper part of the centrifugation vessel, while impurities and bundled tubes accumulated at the bottom (Fig. 1c-2). Optical and electrical analysis confirm a high purity separation of semiconducting SWCNTs (results will be discussed below).

We have also investigated the case in which the SPT initial loading in the tube and the molecular weight of the polyethylenglycol side chain in the PAE polymer were altered. Three acidic aqueous SPT solutions (pH 2.5) with decreasing concentrations were layered on top of each other in the centrifugation vessel (Fig. 1b-3). In this experiment the SWCNT dispersion (pH 2.5) was mixed into the topmost SPT layer prior to loading. The molecular weight of the polyethylenglycol side chain in the PAE polymer was decreased from 1,500 g/mol (PAE1) to 750 g/mol (PAE2) as compared to the previous runs. Under these conditions, we observe after 72 h of WFC, and for the first time, the self-organization of SWCNT sheets of high lateral extension. They can be easily extracted as flexible freestanding high purity semiconducting sheets with a diameter in the centimetre range and thickness of about 100 nm (Fig. 1c-3). Before presenting the optical and electrical investigations on the separated band and the extracted freestanding sheets, we will focus in the next section on understanding the separation mechanism and the different results depending on the process conditions applied.

Clearly, the acidity of the heavy liquid strongly influences the outcome of the SWCNT separation. The separation results after systematic pH variation are shown in Fig. 2a. Here, 0.5 ml of a SWCNT dispersion at pH 4 were top-loaded onto 4 ml SPT columns with their pH values varying between 1 and 6. Figure 2b shows the corresponding normalized UV-Vis-NIR absorbance spectra of the extracted and purified top bands (red boxes in Fig. 2a). At pH 1 **a**ll nanotubes sediment and no separation is observed. Apparently, at very low pH values the stabilization of the SWCNTs is fully lost. In fact, electrophoretic mobility measurements on HiPco CNTs dispersed with the PAE1 polymer reveal a strong decrease in the surface charges below pH 3 (Supplementary Section 2). This leads to lower electrostatic repulsion and thus the formation of larger SWCNT bundles and aggregates. As a consequence of the increase in hydrodynamic size and density these bundles move to the bottom of the tube even at low centrifugal fields. For pH values between 4 and 6, the centrifugation stage only leads to a separation of the individualized, but electronically unsorted species of SWCNTs from the carbonaceous impurities in the raw dispersion. Obviously, SWCNTs of all electronic types are equally stabilized under these conditions. Moreover, at this low centrifugal field the formation of an equilibrium density gradient is not expected either. That is why banding, i.e. separation according to chiralities, is not observed^25^. Only the absorbance spectra for the separated fractions at intermediate pH levels 2 and 3, show a decreased absorbance in the metallic M11 transition region^26^ (red shaded area in Fig. 2b). This is a clear indication for the enrichment with semiconducting SWCNTs. In fact, under these conditions a selective protonation of the sidewalls of metallic carbon nanotubes compared to semiconducting SWCNTs is predicted^27^. Consequently, the PAE dispersant predominantly stabilizes semiconducting SWCNTs while partially detaching from the significantly protonated metallic ones that hold fewer adhesion sites available^28^. The agglomeration and sedimentation observed at lower pH values is obviously under these conditions mainly affecting the metallic nanotubes and not their semiconducting counterparts. Moreover, even at such low centrifugal fields we achieve excellent spatial separation between the semiconducting band and the rest of the sample. In contrast to most common separation media, the low viscosity heavy liquid (SPT)^29^ can rapidly form dynamic density gradients in WFC. The non-toxic, recyclable SPT enables high purity fractionation at low g-forces, while stronger centrifugal fields decrease the time to reach the separation state. In this state, the well dispersed semiconducting nanotubes (lower density) are withheld at the upper part of the centrifugation vessel due to the formation of a density barrier^30^ as further explained in Supplementary Section 3.

The detailed understanding of the polymer-SWCNT interaction and its pH dependency is still under investigation and is beyond the scope of this work. Nevertheless, a video is found in the Supplementary material (Supplementary Video 1) showing the onset of agglomeration in the case of unsorted SWCNTs versus a stable dispersion of semiconducting SWCNTs at low pH levels (Supplementary Section 4). In order to visualize agglomeration and sedimentation we have set the pH value of the dispersed SWCNTs to 1. In this regime both SWCNT dispersions are not stable. The video shows the first 40 minutes after the pH value was adjusted. Finally the complete agglomeration of both fractions was only observed after 5 hours, indicating a different susceptibility to agglomeration for both dispersions.

Additionally, stable semiconducting SWCNT fractions separated as discussed before and an enriched metallic SWCNT fraction obtained from the bottom of the centrifugation vessel were used for X-ray photoelectron spectroscopy that can be found in Supplementary Section 5, which further support the hypothesis of PAE detachment from the metallic SWCNTs.

The observation of self-organized freestanding sheets under WFC conditions may be surprising at first glance (Fig. 1c-3). For this experiment the three layered SPT solutions were set to pH 2.5. Clearly, the semiconducting-metallic SWCNTs separation mechanism can be rationalized by taking into account the results presented in the previous paragraph. The formation of the sheet-like structure is believed to be favoured by an increased spatial compression in the centrifugation vessel. In fact, the SPT concentrations of the two top layers correspond to densities of about 1.09 g/cm^3^ and 1.36 g/cm^3^, respectively. The buoyant density of the polymer-SWCNT pair is expected to be within this range^31^. Denser bundles and aggregates may easily penetrate this density interface and precipitate towards the bottom of the tube. The stabilized semiconducting SWCNTs, however, will accumulate at this interface. It is important to mention that the molecular weight of the used PEG side chains was decreased to 750 g/mol. Thus, the degree of steric stabilization is reduced and will further facilitate the formation of these condensed sheet-like structures.

Next, the purity and quality of the separated fractions of semiconducting SWCNTs were analysed using UV-Vis-NIR absorption and Raman spectroscopy. Figure 3a shows the normalized absorbance spectra of three SWCNT samples separated as described in Fig. 1, i.e. under neutral (pH = 7, fraction 1) and acidic conditions (pH = 1.8, fraction 2) as well as the freestanding SWCNT sheet after its transfer to a quartz substrate. All spectra were background corrected to eliminate the contribution of their respective environment (surfactant solution or support). Both absorbance spectra of fraction 2 and of the sheet show a strong decrease in the absorbance in the metallic M11 transition region (~400–600 nm) compared to the absorbance of fraction 1. However, the absorbance spectra of both semiconducting nanotube enriched samples are not identical between 900 nm and 1,100 nm. These differences indicate a slightly different enrichment with respect to certain semiconducting chiralities/diameters for fraction 1 and 2, respectively. Nevertheless the strong suppression of the absorption in the metallic region confirms the advanced electronic type separation of our new method. The exact quantification of the semiconducting purity on the basis of absorbance spectra as suggested in^32^ is difficult for HiPco SWCNTs as detailed in Supplementary Section 6.

Raman spectroscopy was performed to further investigate the quality of the separated SWCNT fractions. The Kataura plot in Fig. 3b relates optical transitions of nanotubes to their diameter or radial breathing mode (RBM), respectively. It has to be noted, that single wavelength excitation Raman spectroscopy gives insight into only a small fraction of SWCNT chiralities. Two excitation wavelengths, at 633 nm and 514 nm, were used to evaluate the SWCNT species^33^,^34^,^35^. The RBMs of all collected fractions excited at the given wavelengths are shown in Fig. 3c,d. Being in line with the previous observations on this sample, fraction 1 reveals strong RBMs in both the metallic and semiconducting regime. In contrast, for fraction 2 (pH 1.8) only RBMs associated with semiconducting SWCNTs were excited. These findings underline once more the highly efficient separation by electronic type under the experimental conditions described above. In the case of the freestanding sheet semiconducting RBMs were excited at both laser wavelengths, but peaks originating from metallic RBMs were also detected when excited by the 514 nm laser. The observation of metallic RBMs in the sheet indicate the presence of residual metallic tubes which were sequestrated in the freestanding sheet. This might indicate that at pH 2.5 the separation of semiconducting from metallic SWCNTs is not complete even after long centrifugation times. These findings might be further influenced by the diameter distribution of the nanotubes observed in the freestanding sheet, which is shifted to smaller diameter SWCNTs compared to fraction 2^27^.

In the last part of this study, we now proceed to the study of the electronic behaviour of devices based on the separated SWCNT material. The two most important figures of merit of FETs are the charge-carrier mobility μ and the ratio between the current passing through the transistor in the on and the off state (I_on/off_)^36^. The lateral dimensions of such FETs are typically in the >10 μm range, meaning that the channel does not consist of a single but a percolating network of a large number of SWCNTs. Such a network naturally exhibits a high amount of junctions between individual SWCNTs that limit μ^37^. The resistance of each junction critically depends on the chemical purity of the sidewalls of the SWCNTs. The removal of the surfactant used for separation or dispersion of carbon nanotubes is very important. In a FET composed of a SWCNT-network, μ and the on/off ratio also correlate with the SWCNT density in the channel. The larger the total density of tubes, the larger also μ, since more tubes contribute to the overall current. At the same time, however, the on/off ratio will typically decrease, as the density of residual metallic SWCNTs increases. In the worst case such high densities may lead to a shortening of the channel and to a decreased on/off ratio. It follows, that a good approach to investigate the degree of purity for separated SWCNTs is to deposit a very dense network as semiconducting channel.

To demonstrate the applicability of our SWCNTs, we have realized field-effect transistors from fraction 2 on aluminium oxide coated silicon wafers. Before deposition on the wafer the SWCNTs were first separated from residual SPT and excess polymer (sample conditioning) and then re-dispersed in sodium deoxycholate hydrate (SDOC)^38^. The layout of a transistor, an atomic force microscope (AFM) picture of the thin-film morphology as well as the input and output curves are shown in Fig. 4. A top-contact bottom-gate structure was used for the device testing (Fig. 4a). The AFM im**a**ge (Fig. 4b) shows that the network exhibits a very high density of SWCNTs. Still, the input curves of the transistors (Fig. 4c) show a p-type field-effect transistor with high on/off ratio (10^5^). Even though the AFM analysis indicates that the nanotubes were not completely liberated of the polymer and surfactant by our cleaning protocol the device shows a remarkable field-effect mobility up to 17 cm^2^/Vs comparable to literature results with similar, plain transistor geometry^8^,^13^. The large hysteresis indicates a high density of trapping sites that is typical for this kind of device geometry^39^ and can be overcome by using different dielectric layouts as reported previously^40^. An improved cleaning protocol of the deposited SWCNTs or a more favourable contact geometry could also lead to better injection and improved transistor performance^13^,^41^,^42^,^43^. However, the large charge carrier mobility with concomitantly large on/off ratio reveal that the tubes obtained by our method are suitable as channel material for thin-film transistors for example for use in OLED displays.

We have also prepared FETs based on the freestanding SWCNT sheet (Fig. 5). The sheet used is approximately 100 nm thick and therefore cannot be contacted with conventional gating methods. To measure its electronic properties, we have therefore utilized an ionic gel (IG) as gate dielectric. The ions contained in the gel can penetrate and therefore gate the SWCNTs throughout the entire sheet.

A micrograph (Fig. 5a) shows the layout of the SWCNT sheet device on a quartz wafer. Capacitance measurements, transconductance and output characteristics of the sheet transistor are summarized in Fig. 5b–d. In Fig. 5b the area normalized capacitance of the IG at 1 Hz for different gate-source voltages is found^44^. The complete curve was normalized to the interface between IG and the SWCNT sheet in the on-state. It is found that the transistor shows even for high V_ds_ = −1 V a notable on/off behaviour (10^3^) (Fig. 5c), which is a good indication for the high purity of the SWCNTs given that this transistor device is composed of a 100 nm dense SWCNT network and the channel is only 50 μm in length. The hysteresis is negligible mainly due to isolation of the nanotube channel by the IG and measurement in vacuum^39^. This also leads to the characteristic ambipolar transistor behaviour for SWCNTs as it was reported previously^45^,^46^,^47^. The sheet transistor has a hole mobility of 12 cm^2^/Vs and an electron mobility of 11 cm^2^/Vs calculated by using the peak capacitance for electron (55 μF/cm^2^) and hole (66 μF/cm^2^) conductance in the corresponding voltage range. We believe this mobility can be improved in the future by removing the polymer content from the sheet which remains from the separation stage. The mobility of the transistor is furthermore limited by the carrier injection at the metal contacts (Fig. 5d) due to the top contact top gate geometry applied for the measurements.

In summary, we have demonstrated a highly efficient and simple method to isolate semiconducting species from as-produced SWCNTs. The development of tailor-made polymers with strong amphiphilic character and the usage of low viscosity heavy liquids lead to very high degrees of semiconductor enrichment using conventional centrifugation only. With this method, not only individualized SWCNTs can be isolated but also flexible and extended semiconducting SWCNT sheets are obtainable. The purity and quality of the isolated SWCNT fractions were analysed using UV-Vis-NIR as well as Raman spectroscopy. Promising results from field-effect transistor measurements further confirm the high electronic quality of the purified SWCNTs. Overall, with this new technology a simplified approach towards SWCNT sorting using only standard laboratory equipment is introduced. Throughout the entire process a maximum g-force 10,000 × *g* was required, which enables a transfer of the method without major modifications to large-scale production equipment.

## Methods

### Preparation of SWCNT – dispersion

HiPco SWCNTs were purchased from NanoIntegris (Batch# R1-912). The as-produced HiPco SWCNTs were dispersed in an aqueous solution containing 2 wt% of PAE1. The detailed synthesis of the PAEs can be found in Supplementary Section 1.

The aqueous dispersion was obtained by horn sonication for 1 h at 100% amplitude (Dr. Hielscher UP200s) starting with 0.5 wt% of raw nanotube material in a total of 50 g of dispersion. During sonication the dispersion was placed in ice-cooled water bath. After the material had been dispersed, the pH-value of the dispersion was set to 4 by adding 1 M HCl (Knick pH-Meter 766).

### Centrifugation process

For centrifugation, a water based solution of sodium polytungstate (SPT) (TC Tungsten Compounds) 25.5 wt% also containing 2 wt% of PAE1 was prepared. The pH-value of this SPT column was controlled by adding 1 M HCl. The centrifugation vessel was loaded with 4.2 ml of SPT (pH 1.8) and 0.3 ml of the nanotube dispersion (pH 4) on top. Centrifugation (Beckman Coulter Optima XL) was performed applying a centrifugal field of approximately 10,000 × *g* for 25 h to yield semiconducting nanotubes in a Beckman Coulter SW 60Ti rotor.

In case of the freestanding SWCNT sheets the dispersion was created using PAE2 with shorter PEG sidechains (Supplementary Section 1) (pH 2.5). For centrifugation 7.5 ml of the raw SWCNT dispersion was mixed with 7.5 ml of 20 wt% aqueous SPT solution (pH 2.5). The volume of the centrifugation vessel was changed from a 4.5 ml to a 29.5 ml tube. Two 7 ml columns of 64 wt% and 34 wt% SPT were set up on top of each other. As the topmost layer 15 ml of the SWCNT dispersion/SPT mixture was used. The pH-value of each layer was set to 2.5. Centrifugation was performed applying a centrifugal field of approximately 10,000 × *g* for 72 h in a Beckman Coulter SW 32Ti rotor.

### Post-treatment of the separated SWCNTs and re-dispersion into semiconducting inks

The extracted semiconducting SWCNT fractions that still contained SPT and the PAE1 polymer were slowly mixed with 1 M HCl. This leads to the agglomeration of SWCNTs. The agglomerated particles were collected after 2 h, diluted in DI-water and submitted to a 15 min run in the centrifuge at 10,000 × g. The supernatant was extracted and the pellet was swirled up by refilling the centrifugation vessel with DI-water. This manoeuvre was repeated 6 times. Afterwards the pellet was swirled up in DI-water containing 1 wt% of SDOC (Alfa-Aesar) (pH 7.2). Bath-sonication for 30 min was used to disperse the SWCNTs again.

The SWCNT sheet was collected and stored as obtained in a DI-water bath. A part of the sheet was transferred to a quartz wafer and was subsequently dried at 70 °C.

### Characterization

Optical analysis was performed recording the absorbance spectra of post-treated samples with the Perkin Elmer UV-Vis-NIR Spectrometer Lambda 750 with 1 cm cuvettes and for Raman spectroscopy a ND-MDT Ntegra Spectra was used. The morphology of SWCNT networks was determined by tapping-mode AFM (Bruker Dimension Icon).

### Transistor measurements

For the SWCNT network transistor the conditioned sample was drop cast onto an aluminium oxide/silicon wafer (30 nm dielectric thickness) in single droplet steps. The wafer was heated to 80 °C to evaporate the water content of each drop. After evaporation of the water, the wafer was quickly dipped in 1 M HCl and afterwards placed in an Ethanol bath for 5 minutes. This procedure was repeated 30 times. The wafer was patterned with gold electrodes by vacuum evaporation. Channels of 50 μm length and 200 μm width were established.

The characteristic curves were recorded with an Agilent B1500 Parameter Analyzer. The saturation mobility was calculated using the formula 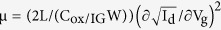 using the plate-plate capacitance model for the network transistor.

A part of the SWCNT sheet was deposited from a water droplet onto a quartz wafer and was subsequently dried at 70 °C. Metal contacts were realized via thermal evaporation of gold through a shadow mask and formed a transistor channel of 1,000 μm width and 50 μm length. The IG was formed from a mixture of 1-ethyl-3-methylimidazolium bis(trifluoromethylsulfonyl)imide ([EMIM][TFSI], BASF SE), poly(vinylidene-co-hexafluoropropylene) (p(VDF-HFP), Sigma Aldrich) and γ-butyrolactone (Sigma Aldrich), respectively and dried onto the sheet at 70 °C and 5 mbar. A PEDOT:PSS gate electrode was drop-cast on top of the IG and dried for 30 min at 70 °C to allow a better electrical contact between the IG and a probe needle.

## Additional Information

**How to cite this article**: Reis, W. G. *et al.* Highly Efficient and Scalable Separation of Semiconducting Carbon Nanotubes via Weak Field Centrifugation. *Sci. Rep.* **6**, 26259; doi: 10.1038/srep26259 (2016).

## Supplementary Material

Supplementary Video

Supplementary Information

## Figures and Tables

**Figure 1:**
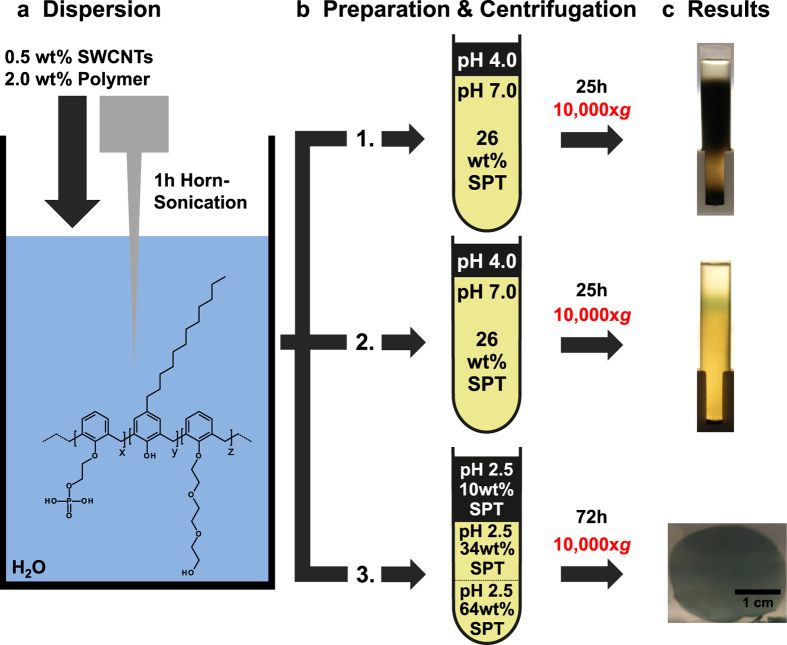
The separation process is shown schematically. (**a**) Raw HiPco SWCNTs are dispersed in an aqueous solution of the PAE dispersant. The general structure of the PAEs is shown. The exact compositions of the PAEs are detailed in the Supplementary Information. (**b**) The neutral (1.), acidic (2.) and acidic layered (3.) preparation of the separation medium and dispersion prior to centrifugation. (**c**) Photographs of the tubes after centrifugation at 10,000 × *g* reveal the separation of electronically unsorted SWCNTs under neutral (1.) and semiconducting SWCNTs under acidic (2.) conditions during centrifugation. The freestanding SWCNT sheet (3.) is shown after extraction from the centrifugation vessel and transfer into a DI-water bath.

**Figure 2:**
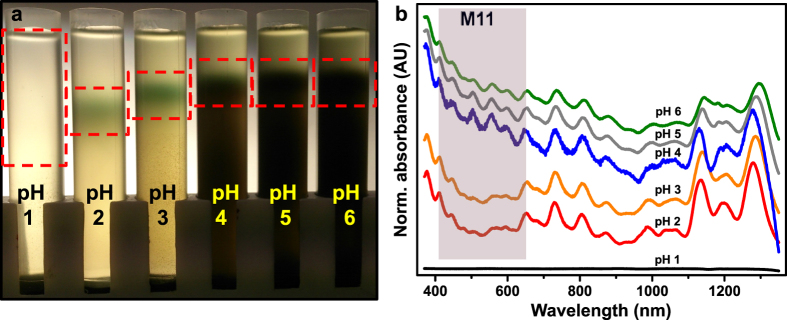
The pH dependency of the weak field SWCNT separation. (**a**) Photograph of the resulting separation of SWCNTs from the raw dispersion after 32 h of centrifugation at 10,000 × *g* for increasing pH-values. (**b**) Normalized (to peak at ≈1270 nm) UV-Vis-NIR absorbance measurements of the separated fractions (red boxes). The spectrum of the pH 1 fraction was not normalized due to a lack of optical transitions. Spectra were offset into the y-direction for clarity.

**Figure 3:**
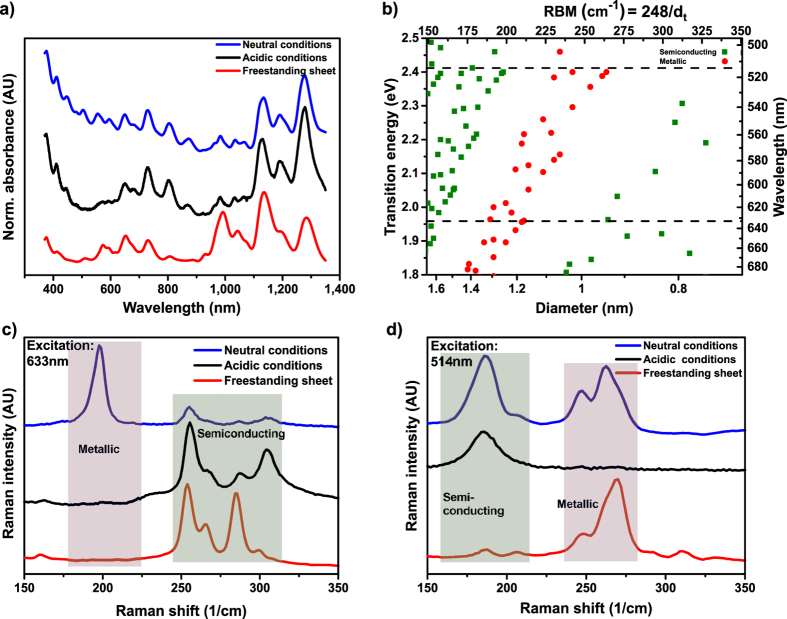
Optical analysis of the separated fractions. (**a**) Normalized UV-Vis-NIR spectra after pH-neutral separation of HiPco SWCNTs (blue), acidic conditions (pH 1.8, black) and the freestanding sheet (pH 2.5, red). (**b**) The Kataura plot was reproduced from calculations following^48^ shown in the diameter range of HiPco SWCNTs. E22 transitions were taken from^49^. Red dots indicate metallic SWCNTs, green dots show semiconducting SWCNTs. The dashed lines in (**b**) indicate the laser wavelengths used for Raman spectroscopy. (**c**,**d**) Normalized Raman signals of SWCNTs obtained at neutral (blue), acidic conditions (pH 1.8, black) and the freestanding sheets (pH 2.5, red) were recorded at 633 nm excitation and 514 nm, respectively. All spectra were offset into the y-direction for clarity.

**Figure 4:**
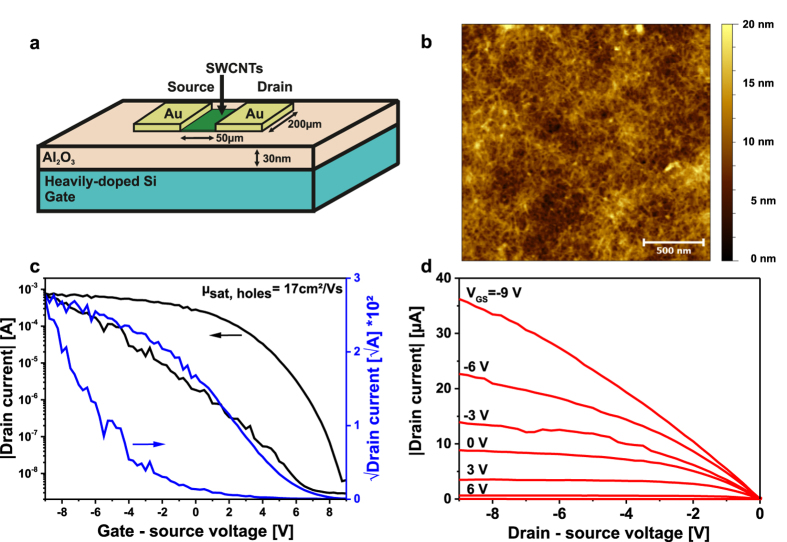
Morphology and electronic characteristics of a SWCNT-network transistor. (**a**) Transistor geometry with semiconducting SWCNT material in green. (**b**) AFM image of the SWCNT network revealing the morphology of the thin film. (**c**) Transconductance (linear fits to √I_D_ resulted in a slope of −0.0028 for the p-region) and (**d**) output curve of a transistor.

**Figure 5:**
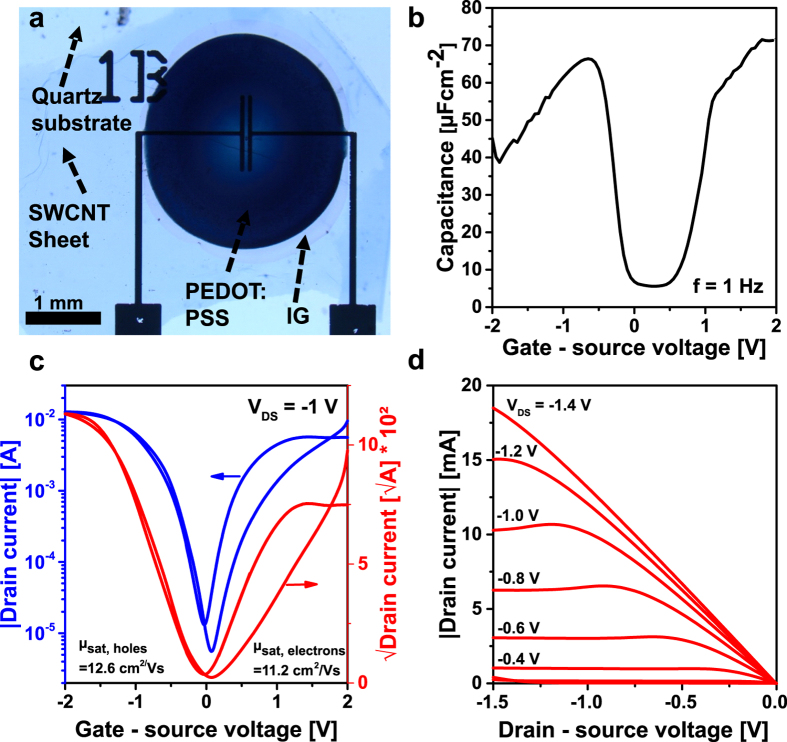
Morphology and electronic characteristics of a SWCNT-sheet transistor. (**a**) Darkfield microscopy image of the sheet transistor covered with IG and PEDOT:PSS (dark blue), SWCNT sheet (intermediate blue) and quartz wafer (light blue). The channel geometry can be seen through the IG. (**b**) Area normalized capacitance of the IG measured at 1 Hz (interface between IG and SWCNT sheet: 7.02 mm^2^). (**c**) Transconductance (linear fits to √I_D_ gave slopes of −0.091 for the p-region and 0,079 for the n-region) and (**d**) output curve of the transistor.
