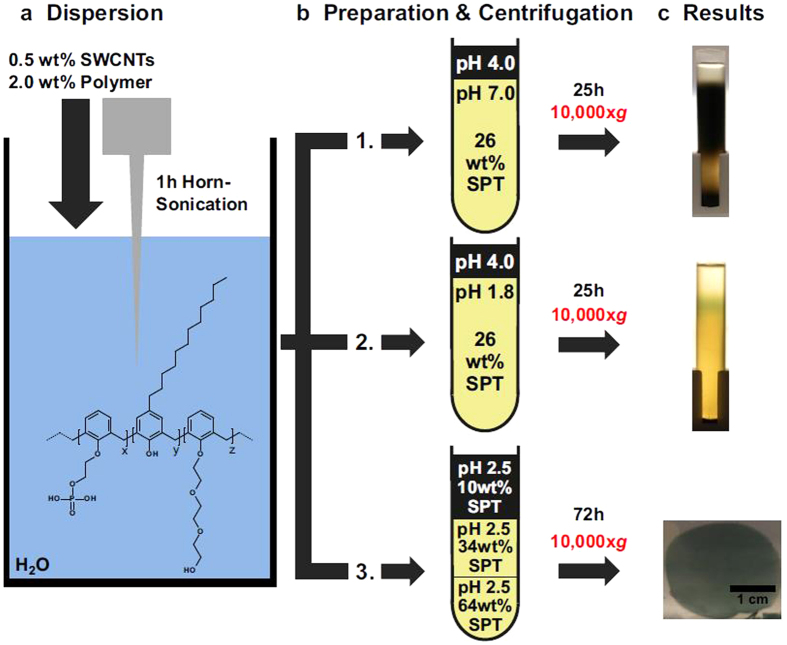# Corrigendum: Highly Efficient and Scalable Separation of Semiconducting Carbon Nanotubes via Weak Field Centrifugation

**DOI:** 10.1038/srep28212

**Published:** 2016-07-20

**Authors:** Wieland G. Reis, R. Thomas Weitz, Michel Kettner, Alexander Kraus, Matthias Georg Schwab, Željko Tomović, Ralph Krupke, Jules Mikhael

Scientific Reports 6: Article number: 2625910.1038/srep26259; published online: 05 18 2016; updated: 07 20 2016

In this Article, there are typographical errors in Figure 1b. In test tube 2, ‘pH 1.8’ is incorrectly given as ‘pH 7.0’. The correct Figure 1 appears below.

## Figures and Tables

**Figure 1:**