# Supplementary material for: Highly Efficient and Scalable Separation of Semiconducting Carbon Nanotubes via Weak Field Centrifugation
Source: Sci Rep. 2016 May 18;6:26259. doi: 10.1038/srep26259 (PMC4870699; doi:10.1038/srep26259)
Supplement: Supplementary Information [file srep26259-s2.pdf]

Supplementary Information

Highly Efficient and Scalable Separation of Semiconducting Carbon Nanotubes via Weak Field Centrifugation

Wieland G. Reis[1], R. Thomas Weitz[2,3], Michel Kettner[2,3], Alexander Kraus[4], Matthias Georg Schwab[1], Željko Tomović[1], Ralph Krupke[5] and Jules Mikhael[6,*]

[1] Carbon Materials Innovation Center (CMIC), BASF SE, 67056 Ludwigshafen, Germany

[2] FET Systems, BASF SE, 67056 Ludwigshafen, Germany

[3] InnovationLab GmbH, Heidelberg, Germany

[4] Advanced Materials & Systems Research, BASF Construction Solutions GmbH, 83308 Trostberg, Germany

[5] Department of Materials and Earth Sciences, Technische Universität Darmstadt, Darmstadt, Germany

[6] Material Physics Research, BASF SE, 67056 Ludwigshafen, Germany

* Corresponding Authors E-mail: jules.mikhael@basf.com

1. Synthesis of Polyarylethers (PAEs)

The PAEs (**Supporting Fig. 1**) were prepared according to the following general phosphorylation and polycondensation procedure:

A reactor, equipped with heating and stirrer, was charged with 127 g of polyphosphoric acid (specified to have 85 % P_2O_5 content) and heated to 90 °C. 138.2 g (1 mol) of phenoxyethanol was added to the stirred reaction mixture through a period of one hour. After the addition was finished, the reaction mixture was stirred for an additional hour. The reaction product contained 75 wt% of phenoxyethanol phosphoric acid monoester (ester of 1 mol of phenoxyethanol with 1 mol of phosphoric acid), 5 wt% of bis(phenoxyethanol)phosphoric acid ester (ester of 2 mols phenoxyethanol with 1 mol of phosphoric acid), 1 wt% of unreacted phenoxyethanol and 19 wt% of unreacted phosphoric acid. The reaction product of the phosphorylation was used without further purification as starting material for the following polycondensation step.

General polycondensation procedure:

A corrosion-resistant reactor equipped with a stirrer and temperature control was charged with the starting materials listed in **Supporting Table 1** in the given order:

1. poly(ethylenoxide)monophenylether (Ph-PEG), 2. phosphorylated phenoxyethanol (PPE), 3. dodecylphenol, 4. paraformaldehyde or trioxan, 5. methansulfonic acid 98 %.

Upon completion of the addition of the acid, the reaction mix was heated to 95-100 °C. After 2 hours (for PAE1) and 3 hours (for PAE2) respectively the polycondensation reaction was finished and water was added. The solid content of obtained water solution

of polymer PAE2 was 49.8 wt%. The polymer PAE1 was neutralized with NaOH to pH 7, and the solid content of the product was adjusted with water to 36.2 wt%.

Polymer	Ph-PEG		PPE[1]	Dodecyl-phenol	Trioxan	Paraform-aldehyde	CH_3SO_3H 98%	M_w[2] Reaction product
	M_w [D]	[g]	[g]	[g]	[g]	[g]	[g]	[D]
PAE1	1500	60.7	87.20	20.40	0	16.33	2.39	8,000
PAE2	750	37.51	54.55	13.51	9.03	0	2.92	6,900

Supporting Table 1. Starting materials used for the synthesis of the PAEs. The exact amounts of the starting materials used for the synthesis of polymers PAE1 and PAE2 are listed.

(1) contains 75 wt% of phenoxyethanol phosphoric acid monoester, 5 wt% of bis(phenoxyethanol)phosphoric acid ester, 1 wt% of phenoxyethanol and 19 wt% of phosphoric acid.

(2) The molecular weights of the polymers were determined by using gel permeation chromatography method (GPC). Column combination: OH-Pak SB-G, OH-Pak SB 804

HQ and OH-Pak SB 802.5 HQ by Shodex, Japan; eluent: 80 Vol.% aqueous solution of HCO_2NH_4 (0.05 mol/l) and 20 Vol.% acetonitrile; injection volume 100 µl; flow rate 0.5 ml/min. The molecular weight calibration was performed with poly(styrene sulphonate) standards for the UV detector and poly(ethylene oxide) standards for the RI detector. Both standards were purchased from PSS Polymer Standards Service, Germany. In order to determine the molecular weight of the polymers, result based on UV-detection (254 nm) was used, because the UV detector is only responsive towards the aromatic compounds and neglects inorganic impurities, that otherwise could falsify the results for the molecular weights.

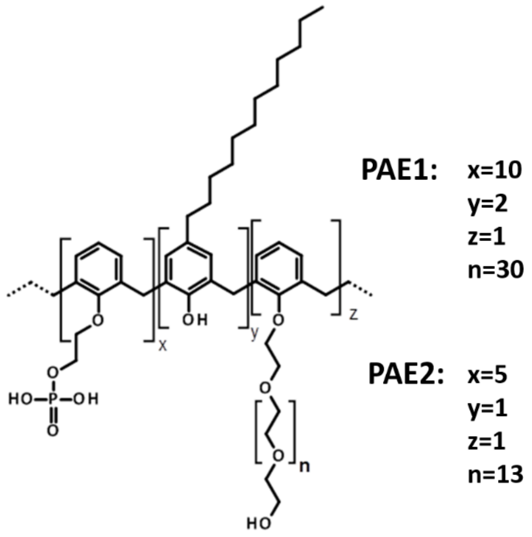

Supporting Fig. 1. Chemical structure of the used PAEs for electronic enrichment of SWCNTs. The general structure of the PAEs used for the separation of SWCNTs. The average compositions for the PAEs are denoted individually. The length of the PEG component is altered besides differences in the component ratio for PAE1 and PAE2, respectively.

2. Electrophoretic mobility

Single-walled carbon nanotubes (SWCNTs) dispersed with PAE1 were submitted to density gradient ultracentrifugation (DGU) at neutral pH to remove all carbonaceous impurities from the raw dispersion. 0.5 ml of the PAE1 nanotube dispersion (set to pH 4) was loaded on top of a column (4 ml of 25.5 wt% of SPT at pH 7) and centrifuged at 40,000 x *g* for 10 h. After extraction of the SWCNTs they were pelletized by centrifugation in water, rinsed with DI-water and re-dispersed in an aqueous solution of PAE1 (2 wt%). The electrophoretic mobility was subsequently measured (Supporting Fig. 2). A decrease in electrophoretic mobility was observed below pH 3 corresponding to a decrease in surface charges.

All measurements were carried out with the Zetasizer Nano from Malvern in pH-range from 2 to 11. The measurements were started at the original pH-value after dilution (7.3). The pH-value was adjusted with HCl and NaOH. All samples were measured at background electrolyte concentration of 10 mmol/l KCl.

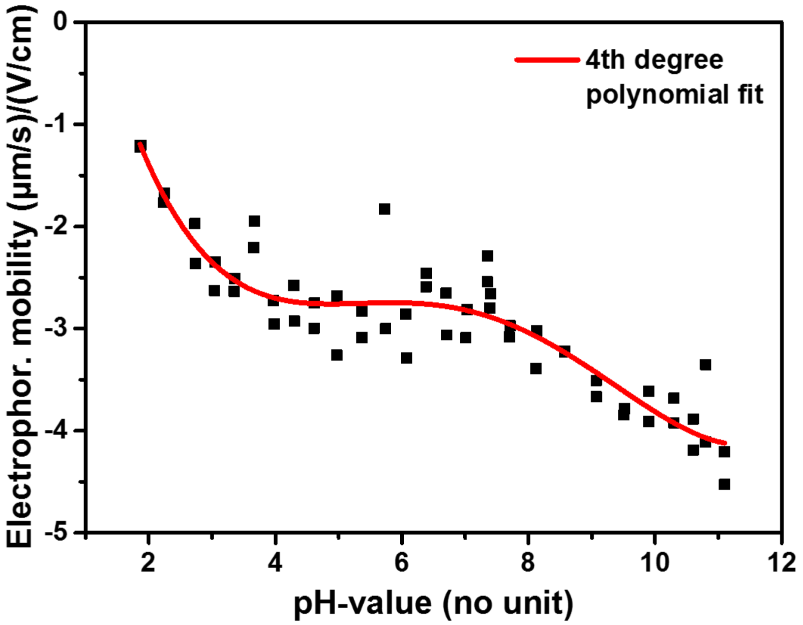

Supporting Fig. 2. Electrophoretic analysis of SWCNTs dispersed with PAE1. The electrophoretic mobility was measured for different pH-values. The electrophoretic mobility decreases significantly in the critical pH-region below 3, where electronic type separation is performed. A fourth degree polynomial is fitted to the data to indicate the trends.

3. Non-equilibrium gradient formation in weak centrifugal fields

Aqueous solutions of common density media were prepared to illustrate the differences between sodium polytungstate [1] (SPT, TC Tungsten Compounds), Caesium chloride (Alfa-Aesar), Iohexol (Axis-Shield) [2] and Iodixanol (Axis-Shield) [3] in weak field centrifugation (WFC). For comparison the density of each aqueous solution was set to approximately 1.24 g/cm^3. Each solution was then centrifuged (Beckman Optima XL) for

11 h at 40,000 x g in a SW60 Ti rotor. Afterwards each centrifugation vessel was separated into 15 fractions, diluted and the density was measured with an Anton Paar DMA 5000M. The density profiles for each separation medium are found in **Supporting Fig. 3**. Ultracentrifugation as well as density measurements were carried out at 25°C.

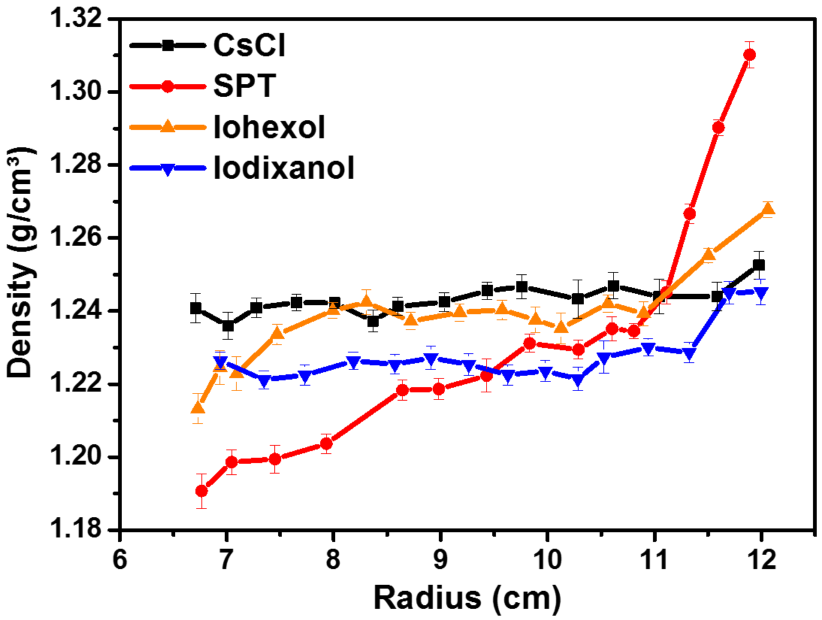

Supporting Fig. 3. Analysis of density gradient formation in WFC. The density gradient profiles of different media at approximately the same initial loading density (1.24 g/cm^3) after centrifugation at 40,000 x *g* for 11 h. The aqueous solution of CsCl does not show density gradient formation at all. Iohexol and Iodixanol exhibit slight density gradient formation. SPT on the other hand shows the most pronounced density gradient formation of the tested media. Error bars indicate the experimental error from dilution of the pristine samples.

4. Video of agglomerating SWCNTs

The SWCNT fractions in the video were collected from neutral and acidic WFC in SPT. As displayed in the main text, WFC under pH neutral conditions leads to the separation of electronically unsorted SWCNTs, while acidification of the SPT column in WFC leads to semiconducting SWCNTs. The extracted fractions were set to pH 1.0, where both fractions are not stable. **Supporting Video 1** shows a time-lapse of the first 40 minutes after acidification of unsorted and semiconducting SWCNTs, respectively. The video is merged from two 20 min videos and sped up by a factor of 64. Within this timeframe agglomeration is starting and the ensuing sedimentation of the agglomerates can be observed for the unsorted SWCNTs while the enriched semiconducting fraction remains stable. Both fractions were found to have agglomerated and precipitated after 5 hours. A different timescale for agglomeration of both heavily acidified samples indicates how the separation mechanism can be understood. At pH 1.8 SWCNTs from the unsorted dispersion begin to agglomerate while the semiconducting nanotubes agglomerate last or even remain stabilized. The increase in density caused by decreased polymer stabilization [4] and the increase in particle sizes due to the following agglomeration enables rapid sedimentation [5] even in WFC. The video was recorded using a Nikon D3200.

5. Selective PAE adsorption on metallic and semiconducting SWCNTs

To obtain fractions enriched with semiconducting or metallic SWCNTs for this experiment, larger agglomerates and bundled SWCNTs were removed from the initial

raw dispersion (PAE1) by a 15 min ultracentrifugation run at 250,000 x *g* and subsequent collection of the supernatant. Samples enriched with semiconducting and with metallic SWCNTs were collected from using the top and bottom fraction after electronic type separation of the obtained supernatant as indicated in **Supporting Fig. 4**. To reveal the electronic character of both SWCNT fractions parts of the top and bottom SWCNT fractions were extracted, set to neutral pH value and centrifuged on a neutral 40 wt% Iohexol gradient with 2 wt% PAE1 at 250,000 x *g* for 18 h. The individualized SWCNT fractions were extracted, diluted with water, pelletized by ultracentrifugation (4h, 250,000 x g), rinsed with DI water and afterwards re-dispersed in 2 wt% aqueous sodium cholate. The obtained samples were analysed by UV-Vis-NIR **(Supporting Fig. 4)** to reveal the corresponding electronic characters of both SWCNT fractions.

The other part of the electronically enriched top and bottom fractions of SWCNTs were analysed by X-ray photoelectron spectroscopy (XPS) to investigate differences in adsorption of the PAE1 dispersant. For that purpose, both as-extracted electronically enriched SWCNT fractions were diluted in DI-water, pelletized by ultracentrifugation at 250,000 x *g*, wet deposited on a silicon wafer and dried for 3 weeks in a vacuum oven (55°C). The raw HiPco SWCNT material was used as reference material. The XPS measurements showed for each sample approximately 80% of carbon, however, the atomic concentration distributions were normalized to the carbon content to minimize the influence of sample abundance. The normalized measurements are summarized in **Supporting Fig. 5**. Phosphorous is an element that can be unambiguously ascribed to the PAEs (here PAE1, see **Supporting Fig. 1**). The normalized atomic concentrations

reveal that the relative amount of phosphorous within the enriched metallic SWCNT fraction is lower than the phosphorous content within the enriched semiconducting sample. Also, the raw reference material shows no phosphorous content. This reveals that the polymeric coating of metallic SWCNTs with PAE is at least partially removed by the acidic environment. The reference material also reveals remaining iron catalyst from the production process. The iron is removed by the separation process in the ultracentrifuge as evident by the lack of the iron signal in the separated samples.

The XPS analyses were carried out with a Phi Versa Probe 5000 spectrometer using monochromatic Al Kα radiation (49 W).

XPS can detect all elements except hydrogen and helium, probes the surface of the sample to a depth of 5-7 nanometres, and has detection limits ranging from 0.1 to 0.5 atomic percent depending on the element. The instrument work function was calibrated to give a binding energy (BE) of 84.00 eV for the Au $4f_{7/2}$ line of metallic gold and the spectrometer dispersion was adjusted to give a BE of 932.62 eV for the Cu $2p_{3/2}$ line of metallic copper.

The built in Phi charge neutralizer system was used on all specimens. To minimize the effects of differential charging, all samples were mounted insulated against ground.

Survey scan analyses were carried out with an analysis spot of 100x1400 μm^2 area, a pass energy of 117 eV and an energy step size of 0.5 eV. High resolution analyses were carried out on the same analysis area with a pass energy of 23.5 eV and an energy step size of 0.1 eV.

Spectra have been charge corrected to the main line of the carbon 1s spectrum set to 284.5 eV as a typical value quoted for the energy of the peak of aromatic carbon.

C1s-Spectra were analysed using CasaXPS (Casa Software Ltd) software version 2.3.16 using Shirley background subtraction in the energy region of 280-298.5 eV. Sp^2-hybridized carbon was described by a Lorentzian asymmetric line shape with tail damping as provided by the software with an asymmetry index of 0.0913 allowed to vary its FWHM in the range of 0.7 to 1.3 eV. All other peaks were fitted using a sum function of 90%-Gaussian-10%-Lorentzian as provided from the software accounting for the presence of functional groups (Hydroxyl, carboxyl, Epoxy, as well as resonances from the aromatic system (shake-up structures and plasmon resonances) as given in the literature [6]. Relative sensitivity factors as provided by the instrument manufacturer were used for quantification.

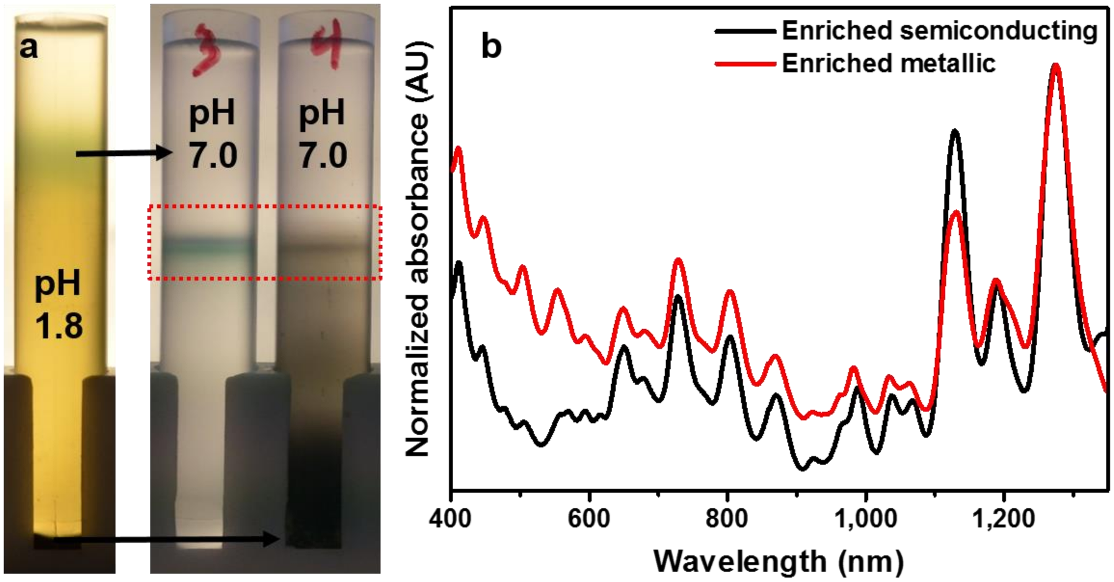

Supporting Fig. 4. Analysis of the fractions used for XPS measurements. a, Parts of the separated top and bottom fractions from electronic sorting were transferred to neutral DGU (40,000 x *g*, 18 h) in Iohexol to isolate the electronically enriched fractions (dashed red box). b, The normalized (peak at 1,275 nm) UV-Vis-NIR spectra reveal the electronic enrichment within each sample. The differences in absorbance in the M11 transition region (400-600 nm) indicate these findings.

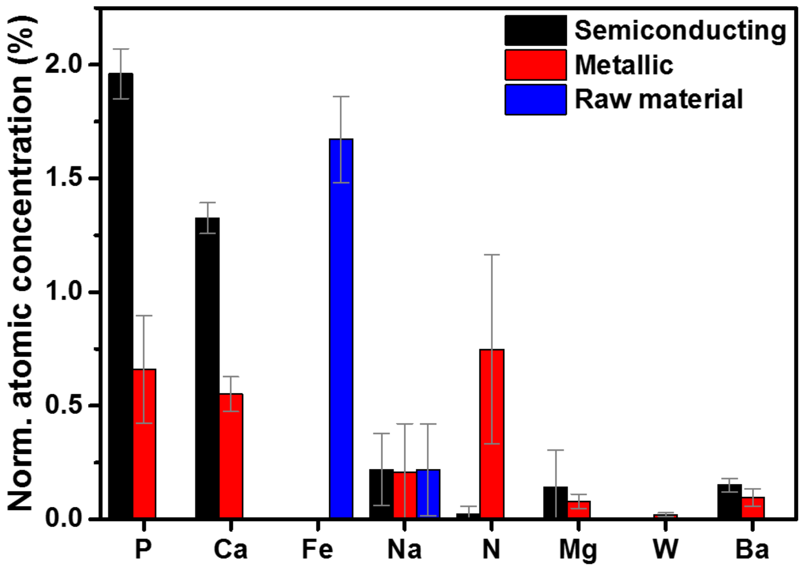

Supporting Fig. 5. Comparing polymeric adsorption on electronically sorted SWCNT fractions after separation. The normalized atomic concentrations of electronically enriched fractions of SWCNTs as well as for the raw material are shown as determined by XPS measurements. The atomic concentrations are normalized to the carbon content. The carbon and oxygen concentrations are not shown to depict the less

abundant elements in detail. Errors bars indicate the statistical variation from measuring multiple spots on each sample.

6. Estimation of semiconducting purity from UV-VIS-NIR spectra

The semiconducting purity of SWCNT samples may be calculated via the UV-Vis-NIR spectra [7]. Therefore the semiconducting S22 and metallic M11 transitions have to be identified. For large diameter SWCNTs (1.5 nm) these two transition regions can be individually detected enabling to calculate the semiconducting purity. For small diameter HiPco SWCNTs (1 nm) the metallic M11 transitions overlap to a larger extend with the S33 and S22 transitions of the semiconducting nanotubes [8, 9]. However, the proposed method requires the peak areas to be identified unambiguously. Thus this method tends to underestimate the semiconducting purity of the fractionated HiPco samples if the overlap area is ascribed to the metallic SWCNTs or might overestimate the semiconducting purity if the overlapping area is ascribed solely to the semiconducting SWCNTs. Yet, we included a purity estimating calculation for the separated SWCNTs (pH 1.8) here. The UV-Vis-NIR spectra (**Supporting Fig. 4**) reveal an area of decreased absorbance between 450 nm and 560 nm. This region was therefore ascribed to the M11 transitions and used to estimate the semiconducting purity.

In order to estimate the semiconducting purity of the separated nanotube fraction from acidic conditions (pH 1.8), the combined area A(S22) of the semiconducting absorption peaks and the combined area A(M11) of the metallic absorption peaks were calculated after background subtraction. The background fit was performed using an exponential

decay ($C + ae^{-\lambda b}$). According to [7] the semiconducting percentage purity (SPP) of enriched semiconducting HiPco fractions can be obtained by the following equation

$$\mathrm{SPP} = \frac{1}{1 + \frac{A(M11)}{1.05 \times A(S22)}}, \qquad (1)$$

where 1.05 is a determined factor for HiPco SWCNTs [7].

The respective areas were calculated to be A(M11) = 2.688 and A(S22) = 59.797. Using these values and equation 1 the SPP was estimated to be 95,9%. This estimation must be handled with caution, since the metallic M11 and the semiconducting S22/S33 transitions cannot be clearly distinguished. For the freestanding sheet, however, the diameter range of the included nanotubes is shifted so that an estimation on the base of HiPco material is not reasonable.

References

1. Plewinsky, B. & Kamps, R. Sodium metatungstate, a new medium for binary and ternary density gradient centrifugation. *Macromol. Chem. Phys.* **185,** 1429-1439 (1984).
2. Backes, C., Hauke, F., Schmidt, C. D. & Hirsch, A. Fractioning hipco and comocat swcnts via density gradient ultracentrifugation by the aid of a novel perylene bisimide derivative surfactant. *Chem. Commun.* 2643-2645 (2009).
3. Arnold, M. S., Stupp, S. I. & Hersam, M. C. Enrichment of single-walled carbon nanotubes by diameter in density gradients. *Nano Lett.* **5,** 713-718 (2005).

4. Arnold, M. S., Suntivich, J., Stupp, S. I. & Hersam, M. C. Hydrodynamic characterization of surfactant encapsulated carbon nanotubes using an analytical ultracentrifuge. *ACS Nano* **2,** 2291-2300 (2008).
5. Maechtle, W. & Börger, L. *Analytical ultracentrifugation of polymers and nanoparticles* (Springer Berlin Heidelberg, 2006)
6. Leiro, J. A., Heinonen, M. H., Laiho, T. & Batirev, I. G. Core-level xps spectra of fullerene, highly oriented pyrolitic graphite, and glassy carbon. *J. Electron. Spectrosc. Relat. Phenom.* **128,** 205-213 (2003).
7. Huang, L. et al. A generalized method for evaluating the metallic-to-semiconducting ratio of separated single-walled carbon nanotubes by uv−vis−nir characterization. *J. Phys. Chem. C* **114,** 12095-12098 (2010).
8. Weisman, R. B. & Bachilo, S. M. Dependence of optical transition energies on structure for single-walled carbon nanotubes in aqueous suspension: An empirical kataura plot. *Nano Lett.* **3,** 1235-1238 (2003).
9. Nanot, S., Hároz, E. H., Kim, J.-H., Hauge, R. H. & Kono, J. Optoelectronic properties of single-wall carbon nanotubes. *Adv. Mater.* **24,** 4977-4994 (2012).

Video Caption

Supporting Video 1. Video of two SWCNT dispersions reacting to acidic environment. The video shows the first 40 minutes after pH alteration (set to pH 1) of two SWCNT dispersions. One is enriched with semiconducting species (left) and one contains electronically unsorted SWCNTs (right). Both samples contain the as extracted amount of SPT according to the density region in the gradient, where the SWCNTs were found. The agglomeration of SWCNTs can be observed over the course of the video for the unsorted fraction.
